# Individual Differences in Response to Ambiguous Stimuli in a Modified Go/No-Go Paradigm are Associated with Personality in Family Dogs

**DOI:** 10.1038/s41598-019-47510-z

**Published:** 2019-07-30

**Authors:** Nóra Bunford, Barbara Csibra, Márta Gácsi

**Affiliations:** 1 Eötvös Loránd University, Institute of Biology, Department of Ethology, Pázmány Péter sétány 1/C, Budapest, 1117 Hungary; 2 Hungarian Academy of Sciences, Research Centre for Natural Sciences, Institute of Cognitive Neuroscience and Psychology, Magyar tudósok körútja 2, Budapest, 1117 Hungary; 3 MTA-ELTE Comparative Ethology Research Group, Pázmány Péter sétány 1/C, Budapest, 1117 Hungary

**Keywords:** Psychology, Animal behaviour

## Abstract

Cognitive biases, often used as indices of affective and emotional states, are associated with individual differences in personality in humans and have been observed in nonhuman animals, including dogs. Although dogs have complementary advantages over traditional animal models of human cognition, little is known about the relationship between dogs’ cognitive bias and personality. Here, we examined in 29 family dogs (representing 14 breeds and 12 mutts; *M*_age_ = 4.59 years, *SD* = 2.90), the association between naturally occurring – as opposed to experimentally induced – cognitive bias, indexed via active choice behavior in a Go/No-Go (GNG) paradigm reflecting positive/negative expectations about ambiguous stimuli, and owner-rated personality. In a subsample we additionally assessed whether prior inhibition, personality, and inattention (IA)/hyperactivity/impulsivity (H/I) results could be replicated in a modified paradigm. We also explored whether expanding the response time-window would increase GNG errors and whether dogs exhibited differences in their behavioral approach to uncertainty. Findings indicated dogs with higher conscientiousness and extraversion scores were more likely to exhibit a “go” response to ambiguous stimuli. Replicability across prior and current results was generally established, e.g., as previously, IA did not predict GNG performance but extraversion did, whereas H/I predicted different indices of GNG performance. Increased response time-window did not result in differential performance, except for less commission errors. No differences in behavioral response strategy to trained “no-go” and to ambiguous stimuli were apparent. Results evince the dog is a promising animal model of the association between an optimistic cognitive bias and personality.

## Introduction

Individual differences in judgments about ambiguous stimuli, i.e., cognitive biases, occur when an affective state or temperamental trait affects cognitive processes^1^. Here, we conceptualize cognitive bias as a bipolar individual difference variable ranging from optimistic (or “optimism”, seeing the glass as half full) at the high end to pessimistic (or “pessimism”, seeing the glass as half empty) at the low end^2^. An optimal level of optimism (falling in-between overly low and overly high levels and defined by its consequences) has beneficial effects (e.g., lowers anxiety and stress, promotes health)^3^, yet, overly high or low levels (i.e., excessive optimism and pessimism, respectively) are associated with negative outcomes^3,4^.

## Cognitive Bias in Nonhuman Animals

Cognitive biases have been observed in a range of nonhuman animal species (hereafter: animals), including chicks^5^, grizzly bears^6^, honeybees^7^, pigs^8^, rats^9,10^, primates^11,12^, sheep^13,14^, starlings^15–17^, cats^18^, and dogs^10,19–21^. As cognitive biases are often used to indirectly index affective/emotional states, animal research on the phenomenon has implications for animal welfare^1,21^. Across the corresponding studies, inner states were experimentally manipulated to induce cognitive bias (e.g., via provision/removal of environmental enrichment^10,15^, variation of lighting conditions^22^, and negative and positive emotion induction^5,9,23,24^, respectively]). Active choice (Go/Go) tasks^20^ and Go/No-Go paradigms (GNG, e.g.,^9,16^) were used to measure the effects of cognitive biases on emotional states and disorders (e.g., depression). For example, in a pioneering study^9^ combining negative emotion induction and a GNG task, rats (*Rattus norvegicus*) were housed either under stable or under unstable conditions (the latter was hypothesized to promote negative affect and thereby pessimism). Rats were then trained to press a lever to the “go” stimulus to obtain a food reward and also to withhold this behavior in response to the “no-go” stimulus and avoid a burst of aversive white noise. When presented with “go” and ambiguous stimuli, i.e., intermediate between “go” and “no-go” training stimuli, rats housed under stable conditions responded more quickly and frequently both to “go” and to ambiguous stimuli compared to animals housed under unstable conditions (indicating less optimism or greater pessimism in the latter group).

### Cognitive bias in dogs

Complementing traditional animal models, the domestic dog (*Canis familiaris*) is a powerful model of certain behavioral characteristics of humans^25^, with particular advantages over e.g., the rodent model^26^. The results of a few canine cognitive bias (simple discrimination task) studies indicate that dogs exhibit individual differences in such bias comparable to that observed in humans^19,20^. These are related to their affectivity/emotionality^19^ and influenced by similar neurohormonal mechanisms as in humans^20^. Others have also suggested that the link between judgement biases and its correlates (e.g., affective states) in animals may be confounded by third variables such as personality^21^.

## Advancing the State of the Science

To advance the animal model research on cognitive bias, prudent next steps involve (1) moving beyond examining the effects of experimentally induced cognitive biases to examine the relationship between *naturally occurring individual differences* in those biases and the correlates thereof and (2) addressing these questions with a species with complementary advantages to traditional animal models, including with regard to greater similarities to relevant human characteristics.

### Cognitive bias and personality (in dogs)

Of interest to the current study, individual differences in cognitive biases in humans are related to dimensions of personality. Associations are observed mainly with regards to greater optimism being related to higher extraversion and lower neuroticism^27–29^. In addition, there is some evidence that greater optimism is related to higher agreeableness, conscientiousness, and openness^2^.

Prior findings support the dog as an animal model of these cognitive bias-related characteristics as dogs exhibit measurable differences in personality (agreeableness, extraversion, neuroticism, openness^30^; agreeableness, conscientiousness, extraversion, neuroticism, openness^31^) and these are related to their behavioral performance on a touchscreen GNG paradigm^32^. Specifically, openness, confidence, and extraversion predict dogs’ average latency to correct “go” responses and to commission errors. Dogs are less likely to have an earlier correct “go” response (i.e., they were slower) if they have lower scores on openness but more likely to have an earlier correct “go” response (i.e., they were faster) if they are higher on confidence or extraversion. Further, dogs are less likely to have an earlier commission error if they have lower scores on openness but more likely to make such an error if they have higher scores on confidence, or extraversion. These findings are generally consistent with corresponding relationships of these variables in humans^32^, which indicate agreeableness and extraversion are negatively whereas neuroticism is positively associated with behavioral inhibition^33^. Other aspects of personality are also linked to differences in inhibition (ideally probed in GNG paradigms): aggression is positively associated with behavioral disinhibition in 5-HT1B serotonin receptor knockout mice^34,35^ and aggression and impulsivity are linked to reduced levels of the serotonin metabolite 5-hydroxyindole acetic acid in mice^36^ and primates^37^.

#### Passive and active choice paradigms to probe cognitive bias in dogs

As reviewed, discrimination tasks are one effective method for probing cognitive bias in animals. However, in case of dogs, in all studies conducted to date, only a specific type of experimental paradigm (i.e., simple discrimination paradigms relying on passive choice), has been employed to examine and probe cognitive bias. We thus chose a different type of paradigm – a GNG task necessitating active choice – to assess the link between cognitive bias and personality. With these different approaches eliciting slightly different aspects of cognitive bias, they may, over time prove better or worse relative to each other in probing cognitive bias but may also prove complementary. Either way, additional studies, such as the current one, are necessary to ultimately determine the relative advantages and limitations of any given experimental paradigm.

## Current Study

### Primary aims

Accordingly, our aims in this study were twofold. First, to examine whether differences in canine personality are related to *active choice* (as opposed to passive hoping/not hoping) behaviors in a modified GNG paradigm reflecting positive/negative expectations about ambiguous stimuli. Based on human findings, we hypothesized that there would be a positive and strong relationship between confidence and extraversion with behaviors consistent with positive expectations about ambiguous stimuli, and a positive but weaker relationship between agreeableness, conscientiousness, openness and optimism.

Second, to determine whether earlier results regarding the association between GNG performance and attention deficit/hyperactivity disorder – like behaviors and symptoms (ADHD-B/S^32^), can be replicated in the same sample, using a modified experimental design (see *Method*, *Modified Canine GNG paradigm*). In that earlier study, our purpose was to examine associations between dogs’ GNG performance and their owner-rated inattention and hyperactivity/impulsivity, accounting for relevant covariates. In addition to findings noted above (i.e., that openness, confidence, and extraversion predict dogs’ average latency to correct “go” responses and to commission errors), results indicated that greater inattention was associated with shorter latency to commission errors, greater hyperactivity/impulsivity was related to greater proportion of commission errors, and that regardless of accuracy, dogs with basic training had shorter response latencies than dogs with no previous training. In relation to our second aim, we hypothesized that these earlier results could be replicated.

### Secondary aims

#### Examining the effects of extended time on errors

In addition to these primary aims, we also had two exploratory aims. First, given that in our earlier study dogs exhibited a relatively narrow range of GNG errors, we aimed to determine, in a subsample, whether expanding the time window between stimulus onset and feedback (from 3 to 5 s) would result in a wider range of errors – we hypothesized that it would.

#### Implementation of an observational coding system to identify behavioral response types

Second, in our earlier study, we observed that dogs exhibited various behavioral responses while they withheld the prepotent action in response to “no-go” stimuli and when they appeared uncertain about how to respond to such stimuli. In the current study, an observational coding system was employed to empirically evaluate the presence of those differences in behavioral response. We generally hypothesized that in response to the novel ambiguous stimuli, dogs would look at their owners more than in response to “go” and to “no-go” stimuli. Our hypothesis was motivated by the following conceptualization: Dogs could interpret and thus respond to ambiguous stimuli either by treating it as a “go” or as a “no-go” stimulus, and thus respond accordingly. In this case, dogs could either look at the feeder or at the stimulus. Alternatively, dogs could be confused by the stimulus and thus look at their owners and/or frequently shift their attention/gaze^38,39^.

## Method

### Participants

Participants were 29 adult family dogs (*M*_age_ = 4.59 years, *SD* = 2.90) of 14 different breeds and 12 mutts (16 females, 20 neutered animals). The sample size of 29 dogs was chosen as this sized sample was sufficiently large in our earlier, highly relevant work, to observe meaningful effects using comparable and multi-method measurement methods^32^ and also to ensure both feasibility in addressing research questions of interest and minimization of participation burden for owners and dogs (who had to participate in several training and testing sessions, see *Procedures* below). Dogs’ training status was indexed as “none” (no training; *n* = 7 dogs), “basic” (basic obedience training; *n* = 12 dogs), “intermediate” (higher level obedience training; *n* = 4 dogs), or “advanced” (IPO Schutzhund, rescue, service, or gun dog exam; *n* = 6 dogs). This variable was conceptualized as reflecting differences in training status.

Exploratory aims were addressed in a randomly selected subsample of 14 dogs (*M*_age_ = 4.36 years, *SD* = 2.56) of 7 different breeds and 5 mutts (7 females, 3 neutered animals). Three had none, 6 basic, 2 intermediate, and 3 advanced training.

Owners and their dogs were recruited through the Department of Ethology participant pool and website, popular social networking sites, and via snowball sampling. All experimental procedures took place at Eötvös Loránd University, Department of Ethology, in a 3 m × 6 m experimental room.

### Procedures

We first describe the procedures, training, and original GNG test followed by a description of modifications in these regards culminating in the modified GNG tests (MD GNG) and two expanded time window GNG tests (original or modified GNG with expanded time window). For an overview of training and testing phases across our earlier and current study as well as corresponding key details, see Table 1.

**Table 1:** Overview of testing and training phases across our earlier and current study with key details.

	32	Current study						
			Aim 1: Associations between personality, and positive/negative expectations about ambiguous stimuli and Aim 2: Replicability (*n* = 29)		Exploratory Aim 1: Does longer time increase errors? (*n* = 14)		Exploratory Aim 2: Are there observable differences in behavioral approach to uncertainty? (*n* = 14)	
Test/Training	GNG Test	*Training*^a^	MD GNG Test	*Training*^b^	Expanded Time-Window GNG Test	*Training*^c^	Expanded Time-Window MD GNG Test	*Training*^d^
Time for responding	3 s	3 *s*	3 s	3 *s*	5 s	3 *s*	5 s	3 *s*
Session	1 session	*X session (80%)*	4 sessions	*X session (80%)*	1 session	*X session (80%)*	2 sessions	*X session (80%)*
Stimuli	Go (12), No-Go (6)	*Go (12)*, *No-Go (6)*	Go (3), No-Go (3), Ambiguous (3-3)	*Go (12)*, *No-Go (6)*	Go (12), No-Go (6)	*Go (12)*, *No-Go (6)*	Go (3), No-Go (3), Ambiguous (3-3)	*Go (12)*, *No-Go (6)*
Stimuli #/session	2*20	X*20	12	X*20	2*20	X*20	12	X*20
Stimuli # in sum	40		48 (12 Go, 12 No-Go, 24 Ambiguous)		40		24 (6 Go, 6 No-Go, 12 Ambiguous)	
Sound feedback	No	*Yes*	Only after Go and No-Go stimuli	*Yes*	No	*Yes*	Only after Go and No-Go stimuli	*Yes*

*Note*. GNG = Go/No-Go; MD = modified; X session = as many sessions as needed to achieve performance criterion for moving on to the next phase, i.e., completing 20 stimuli in 2 subsequent sets with at least 80% accuracy.

^a^First training after the original GNG test to prepare dogs for MD GNG test. ^b^Starting with the second MD GNG test session, before each test occasions, dogs received a refresher training as depicted. Dogs also received this refresher after the fourth test occasion, i.e., in-between the final MD GNG test session and the expanded time-window GNG test. ^c^Dogs received a refresher training after the expanded time-window GNG test, i.e., in-between the expanded time-window GNG test and the first expanded time-window MD GNG test session. ^d^In-between the first and second expanded time-window MD GNG test session, dogs received a refresher training as depicted. Dogs also received this refresher after the second test occasion.

### Original canine GNG paradigm

Procedures, stimuli, training and details of the Canine original paradigm are as follows.

#### Presentation and recording apparatus

**Figure 1:**
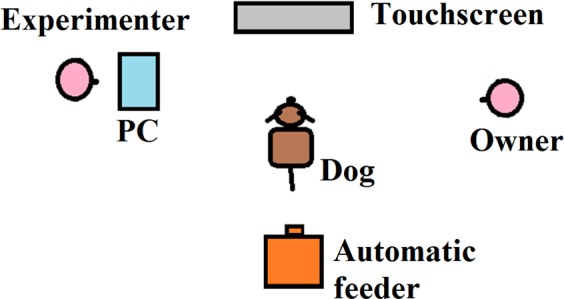
Experimental setup. *Note*. A version of this Figure also appears in (Bunford *et al*., 2018).

Dogs were trained to use a touchscreen device by using their noses to poke or not poke a 36 cm tall and 47 cm wide touchscreen with capacitive sensing to monitor and record touches (31.5 cm × 38.5 cm screen with a 1024 × 768 pixel resolution; ZYTRO-19; Novoparts, Budapest, Hungary), that was mounted to an 82 cm aluminum panel to allow for adjustment to its height. An automatic feeder was placed 2 m away from the touchscreen device. A Windows based PC and the Opensesame 3.0.7 software was used for stimulus presentation and response recording (see^32^ for details and Fig. 1 for experimental setup).

#### Stimuli

Experimental stimuli were blue and yellow circles and triangles (with overall dimensions of 300 × 300 pixels). These colors were chosen as canine cone cells are only sensitive to two colors (i.e., blue and yellow) – thus, those are easiest for dogs to discriminate^40,41^.

As a two-way analysis of variance (ANOVA) examining the main and interaction effects of the two categorical independent variables (color and shape) on dogs’ performance indicated no effects (*p*s > 0.328), dogs were randomly assigned to one of two groups based on stimulus characteristic (color or shape). For one group, a yellow circle was the “go” stimulus and a blue triangle was the “no-go” stimulus whereas for another group, a blue circle was the “go” stimulus and a yellow triangle was the “no-go” stimulus.

#### Training

Training for the GNG test was generally consistent with training used with rodents^42^, except that dogs were also verbally praised and petted. Training comprised three stages, completed on average in *M* = 5.79, *SE* = 0.55 sessions (range: 1–14); *M* = 4.17, *SE* = 0.34 sessions (range: 2–8), and *M* = 8.14, *SE* = 1.05 sessions (range: 1–24), respectively. For details on the training protocol, see^32^.

#### GNG test

In our original Go/No-Go test^32^, dogs were presented with 2 sets of 20 stimuli (60% “go” and 40% “no-go”, with the 60/40 proportion consistent with the literature^43–47^). A correct “go” response indicated that the dog executed a nose poke within 3 s after stimulus onset and an incorrect “go” response, i.e., omission error indicated that the dog did not execute a nose poke within 3 s. A correct “no-go” response indicated that the dog did not execute a nose poke within 3 s after stimulus onset and an incorrect “no-go” response, i.e., commission error indicated that the dog did execute a nose poke within 3 s.

#### MD GNG paradigm

The purpose of the MD GNG paradigm was to address Aims 1 and 2, that is, to (1) examine whether differences in personality and ADHD B/S are related to behaviors reflecting positive/negative expectations about ambiguous stimuli and (2) determine whether earlier results^32^ can be replicated.

Presentation and recording apparatus were identical but stimuli, training, and test details were different across the original and the MD paradigms.

#### Stimuli

In addition to the original, blue and yellow circles and triangles, dogs were also presented with two types of “ambiguous” stimuli, which were different either in color or shape from the previously trained “go” and “no-go” stimuli. Stimulus presentation was semi-randomized so that no more than two stimuli from the same type was presented consecutively.

#### Training

Preparation for the MD GNG test necessitated additional training to ensure that errors are experientially negative. Specifically, although as in the original test, training for the MD test involved 20 stimuli presented in sets of “go” and “no-go” stimuli in a 60/40 ratio, unlike in the original test, in training for the MD test, dogs received feedback both for correct “go” and “no-go” *and* for incorrect “go” and “no-go” responses. Following a correct response, dogs received a food reward *and* heard a “rewarding” (high pitched) sound. Following an omission or commission error, the food reward was withheld, and dogs heard a “punishing” (low pitched) sound. Food reward was withheld but dogs heard no sound following ambiguous stimuli. (Dogs heard a sound not only after correct responses but also after errors so that there is differentiation between feedback to correct and incorrect responses and also between these and feedback following ambiguous stimuli).

Dogs moved on to the MD GNG test after they had completed 20 stimuli in 2 subsequent sets with at least 80% accuracy. On average, dogs completed the training for the MD test in *M* = 3.28, *SE* = 1.13 sessions (range: 2–6). An average of 7.90 days passed (*SE* = 1.95, range 6–12) between this training and the MD GNG test.

#### MD GNG test

In this test, dogs were presented with 4 sessions of 12 trials (25% “go”, 25% “no-go”, and 25% ambiguous (in color) stimulus and 25% ambiguous (in shape) stimulus). Each session was presented on a different occasion, separated by at least one week. Starting with the second test session, before each occasion, dogs received a refresher training session with “go” and “no-go” stimuli to ensure that they would not get overly confused or distracted (and thus demotivated or frustrated) by the lack of reward in case of the ambiguous stimuli.

In the test, dogs had 3 s to respond after stimulus onset and correct and incorrect “go” responses, omission and commission errors were defined as in the original paradigm. In case of the “go” and the “no-go” stimuli, feedback was given in the form of food/no-food and sound as was the case during training for this test. Greater tendency to exhibit a “go” response to ambiguous stimuli was conceptualized as indication of active choice reflecting positive expectations about the stimulus.

#### Expanded time-window GNG paradigm

There were two types of expanded time-window GNG tests. One was identical to the original GNG test, except that instead of 3 s, dogs had 5 s to respond. We refer to this as the expanded time-window GNG test. As in the original test, dogs were tested with 2 sets of 20 stimuli on the same day.

The second was identical to the MD GNG test, except that instead of 3 s, dogs had 5 s to respond. We refer to this as the expanded time-window MD GNG test. As in the MD test, dogs were tested with sets of 12 stimuli on separate days, with at least one week in-between, with training before the second test occasion.

The purpose of the expanded time-window tests were to address Exploratory Aims 1 and 2. In case of the expanded time-window test, the aim was to determine, in a subsample, whether expanding the time window between stimulus onset and feedback (from 3 to 5 s) would result in a wider range of errors. In case of the expanded time-window MD test, the aim was to evaluate individual differences in behavioral approach to uncertainty. Accordingly, behavioral observation and coding were implemented only during the expanded time-window MD test. Of note, different-length time windows may be used when testing individual differences in cognitive bias, depending on the experimental paradigm employed (e.g., 10 s in Starling *et al*.^21^ and 30 s in^19^). Extending the time-window to such lengths would have been impractical. It stands to reason that doing so would have resulted in there being only or almost only commission errors and no or almost no omission errors (though we are not aware of any empirical evidence from other studies that suggests that the longer the time window the more likely an animal will make a commission error) and this is inconsistent with GNG paradigms wherein both correct responses and especially errors (of commission and omission) are performance indices of interest.

All dogs participated in the 3 s GNG test first and then the 5 s GNG tests, i.e., conditions were not counterbalanced, in light of the unreasonable amount of training this would have necessitated and thus attrition this would have caused. As such, the primary goal in comparing performance across the 3 and 5 s tests was to evaluate the presence/absence of any trends towards greater differences in between-dog performance. Thus, the goal was to generate hypotheses to be further evaluated in future studies with a balanced design and perhaps larger samples.

#### Behavioral observation and coding procedures

To assess differences in dogs’ behavioral responses during uncertainty, the expanded time-window MD test was videotaped and coded for predefined behavioral responses, with a 1 s inspection of the recordings using Solomon Coder (© András Péter, http://solomoncoder.com/). Behavioral variables were coded for each time window between stimulus onset (“go”, “no-go”, and “ambiguous”) and the dogs’ response (nose poke) or until 5 s have passed (in case of a withheld response). Although a range of behaviors were examined, other than gaze, other behaviors (e.g., lip licking and vocalizations) occurred with such low frequency that the corresponding data were not analyzable. Thus, behavioral responses of interest were as follows: whether the dog was looking at (a) its owner or the experimenter (looking at person), (b) the feeder (looking at feeder), (c) the touchscreen device/the stimulus (looking at stimulus), or (d) something other than (a)-(c) (looking at other). Direction of gaze or looking was determined by orientation of head and eyes. Inter-rater reliability (Cohen’s Kappa) was calculated for all behavioral variables, by double coding 8 of 28 recordings (29% of the total sample) and indicated almost perfect agreement between the two raters (overall κ = 0.892, looking at person κ = 0.864, feeder κ = 0.921, stimulus κ = 0.928, other κ = 0.848).

### Measures

Owners completed an online questionnaire packet including the following rating scales and questions.

#### Personality

Dogs’ personality was measured using the canine Big Five Personality Inventory^48^, a 43-item owner-report measure, developed based on the Big Five framework, an extensively researched and widely used model of human personality. Owners indicate the degree to which the characteristics described in the items are true about their dog (ranging from ‘not at all’ to ‘extremely’). The measure and its subscales exhibited excellent to acceptable internal consistency^31^. In the current sample, the five subscales, confidence (the opposite of neuroticism) (e.g., “*Is relaxed*, *handles stress well*”, “*Gets nervous easily*”), conscientiousness (e.g., “*Tends to be lazy*”, “*Is a reliable dog*”), cooperation (comparable to agreeableness on the human Big Five) (e.g., “*Is cooperative*”, “*Is generally trusting*”), extraversion (e.g., “*Is full of energy*”, “*Shows a lot of enthusiasm*”), and openness (e.g., “*Is curious about many different things*”, “*Enjoys learning and doing new things*”), exhibited acceptable internal consistency αs ranging from 0.60 to 0.74, except for conscientiousness, which had unacceptable internal consistency α = 0.46).

#### ADHD-B/S

Canine IA and H/I were assessed using the *Dog-ADHD Rating Scale*^49^, a 13-item (6 IA and 7 H/I items) owner-report measure of dogs’ inattention and hyperactivity/impulsivity. The *Dog-ADHD Rating Scale* was developed based on a well-validated and widely used parent-report rating scale of ADHD symptoms and related problems in children, the ADHD-RS-IV^50^. Owners indicate the frequency with which their dog behaves as described in each item (ranging from ‘never’ to ‘very often’). Earlier examinations of the measure’s psychometric properties indicated evidence for its internal consistency^32,49^ and external validity (i.e., age-, sex-, and training-based differences given rating scale scores)^49^. Greater scores indicate greater IA and H/I. In the current sample, the subscales exhibited acceptable internal consistency, with Cronbach’s alphas (α) ranging from 0.60 to 0.88 and so did the total scale α = 0.84.

#### Covariates of non-interest

Relevant covariates that have been previously hypothesized or shown to be associated with differences in canine IA and H/I were dogs’ age, sex, and training status^32,49^.

### Ethical statement

Owners and their dogs participated voluntarily in this research and owners provided written consent. Non-invasive animal research is allowed without need for permission from the University Institutional Animal Care and Use Committee (UIACUC). A statement (#PEI/001/3819-4/2015) indicating that the current study is not considered an animal experiment was obtained from the Food Chain Safety and Animal Health Directorate Government Office, based on the decision of the Scientific Ethic Council of Animal Experiments.

### Analytic plan

All analyses were conducted in SPSS V22.0.0.0. Descriptive statistics were calculated to characterize the sample on all behavioral performance variables. For a list of dependent, independent, and covariate variables, grouped by study aims, see Table 2.

**Table 2:** Dependent, independent, and covariate variables, grouped by aims.

Aims	Dependent variables	Independent variables
Aim 1: Examine whether differences in canine personality is related to behaviors consistent with positive/negative expectations about ambiguous stimuli	- ambiguous “go” % - average latency of “go” responses to ambiguous stimuli	- personality - age^a^ - sex^a^ - training status^a^
Aim 2: Determine whether earlier results regarding the association between Go/No-Go performance and ADHD-B/S can be replicated in the same sample, using a slightly modified experimental design	- omission error % - commission error % - average latency of correct “go” responses - average latency of commission errors	- personality - IA score - H/I score - age^a^ - sex^a^ - training status^a^
	Test variables	
Exploratory aim 1: Determine, in a subsample, whether expanding the time window between stimulus onset and feedback (from 3 s to 5 s) would results in a wider range of errors	- Both from 32 and the current study - omission error % - commission error % - average latency of correct “go” responses - average latency of commission errors	
Exploratory aim 2: Evaluate the differences in behavioral response when uncertain about how to respond to No-Go and ambiguous stimuli	time-percentage of using each behavioral response preceding - “go” responses to ambiguous stimuli, - “no-go” responses to ambiguous stimuli - “no-go” responses to no-go stimuli	

*Note*. ^a^Covariates of non-interest.

ambiguous “go” % = the proportion of “go” response to ambiguous stimuli relative to the total number of ambiguous stimuli; average latency of “go” responses to ambiguous stimuli = the time, in ms, that has passed between ambiguous stimulus onset and execution of a “go” response; omission error % = the proportion of omission errors relative to the total number of “go” stimuli; commission error % = the proportion of commission errors relative to the total number of “no-go” stimuli, average latency of correct “go” responses = the time, in ms, that has passed between stimulus onset and execution of a correct “go” response; average latency of commission errors = the time, in ms, that has passed between stimulus onset and execution of a commission error.

To evaluate the effects of independent and covariate variables on “go” responses to ambiguous stimuli and omission and commission error percent, multivariate linear regression analyses with backward elimination were conducted, taking into consideration both significance level of individual predictors and model fit. To evaluate the effects of independent and covariate variables on latency of “go” responses to ambiguous stimuli and of correct “go” responses and commission errors, survival analyses (i.e., Cox regression analysis with occurrence of a response as terminal event) with backward elimination were conducted, taking into consideration both significance level of individual predictors and model fit. Model assumptions were considered prior to (or following, where appropriate) all analyses; these were met. Results are presented following an estimation, i.e., effect size approach, providing an estimate of an effect size, followed by an exact probability (a *p* value) but no statements about statistical significance and a 95% confidence interval (CI). Results are reported for final set of individual predictors/models only.

For our first exploratory aim, that is, to determine any effects of an expanded time-window between stimulus presentation and feedback on the range of errors, data obtained in^32^ was compared to data obtained in the current study, in 4 paired samples *t*-tests, for the following pairs: omission error percent in^32^ and omission error percent in the current study, commission error percent in^32^ and commission error percent in the current study, average latency of correct “go” responses in^32^ and in the current study, and average latency of commission errors from the earlier and the current study.

For our second exploratory aim, that is, to assess the differences in behavioral responses during uncertainty, dog behavior was coded based on where dogs were looking. Given that this aim was exploratory, in lieu of formal analyses, we calculated, for visual depiction and comparison, during the 5 s time-window following stimulus onset, the time-percentage of time spent using each behavioral response preceding “go” and “no-go” responses to ambiguous stimuli, and time spent using each preceding “no-go” responses to no-go stimuli.

The datasets generated and/or analyzed during the current study are available from the corresponding author upon reasonable request.

## Results

### Descriptive statistics

**Figure 2:**
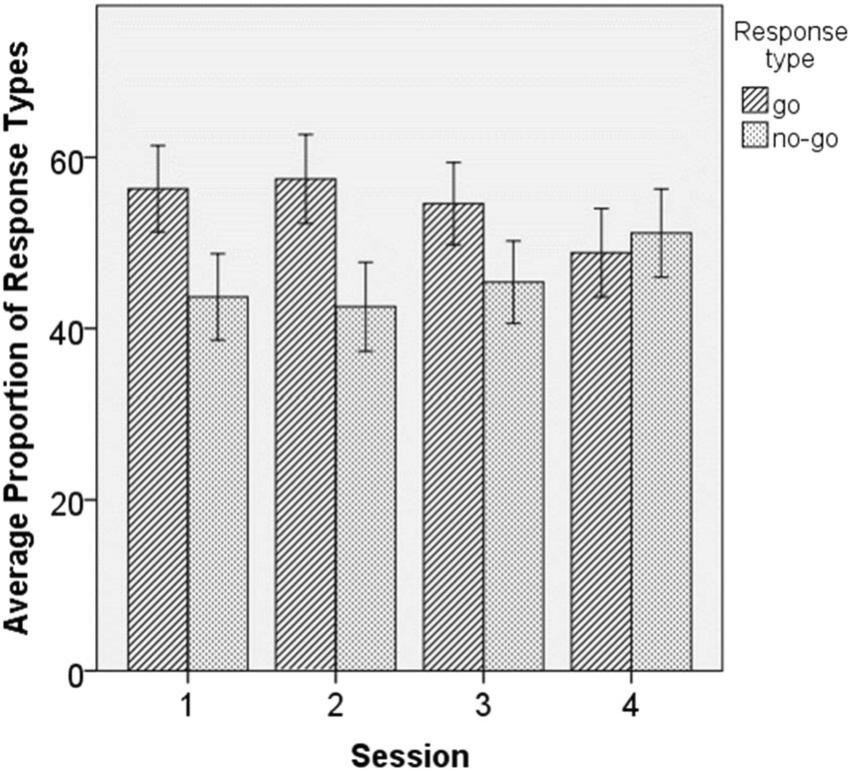
Dogs’ average proportion of “go” (diagonal stripes) and “no-go” (dots) responses to ambiguous stimuli relative to the total number of ambiguous stimuli, presented for each session. *Note*. Error bars represent 1 SE of the mean.

For data on individual dogs across variables of interest, see Table 3 and for descriptive statistics, including *M*, 95% confidence intervals (CI) and range, see Table 4. For results on responses to ambiguous stimuli for each of four sessions, see Fig. 2, which suggests no overall between- or within-session differences in dogs’ tendency to respond to ambiguous stimuli with a “go” or a “no-go” response.

**Table 3:** Data on Individual Dogs Across the canine MD Go/No-Go Behavioral Inhibition Test Performance Variables.

Name	OE%	CE%	Glat (ms)	Clat (ms)	Ambig go%	Ambig go lat
Akina	0,00	16,67	1593,58	3000,00	87,50	1362,56
Alma	0,00	0,00	802,58	3000,00	70,83	1054,50
Barkus	50,00	0,00	1169,38	3000,00	25,00	1011,75
Bingó	0,00	8,33	1164,75	718,00	91,67	989,23
Bogyó	8,33	16,67	1412,08	817,50	45,83	1096,83
Borisz	25,00	8,33	1585,21	266,00	41,67	1827,54
Demi	8,33	0,00	1046,50	3000,00	29,17	1762,00
Dolores	8,33	0,00	1057,54	3000,00	20,83	1330,42
Döme	16,67	16,67	1150,38	1472,50	62,50	1259,03
Joker	8,33	0,00	973,96	3000,00	70,83	1338,80
Kitty	8,33	0,00	875,04	3000,00	66,67	1139,55
Kópé	8,33	0,00	739,58	3000,00	41,67	794,75
Leia	8,33	33,33	1506,33	1636,50	66,67	1493,81
Lili	25,00	16,67	963,88	620,50	41,67	1277,71
Liza	25,00	25,00	1227,04	573,25	45,83	1193,19
Lizi	0,00	16,67	1233,50	1996,50	75,00	1168,23
Lord	0,00	0,00	1058,50	3000,00	37,50	1075,83
Lucky	0,00	16,67	1214,50	2367,00	33,33	1264,25
Mara	41,67	0,00	871,38	3000,00	54,17	1125,89
Molly	25,00	8,33	1171,71	799,00	79,17	1084,45
Monty	41,67	0,00	1315,79	3000,00	8,33	1817,00
Öre	25,00	25,00	906,88	1189,00	54,17	1101,35
Pille	8,33	25,00	911,13	1108,25	45,83	989,38
Rozi	0,00	16,67	1203,00	1336,50	70,83	1650,84
Rynn	8,33	25,00	1282,88	1681,67	54,17	1738,60
Simon	0,00	16,67	826,17	961,00	62,50	1187,84
Vackor	50,00	16,67	1635,88	1157,00	54,17	1531,23
Zajec	16,67	8,33	709,88	470,00	66,67	791,78
Zebulon	33,33	16,67	779,63	809,00	70,83	1130,71

*Note*. OE = omission error; CE = commission error; Glat = latency to correct go responses; Clat = latency to commission errors; Ambig go = “go” response to ambiguous stimulus; Colordiff go = “go” response to ambiguous stimulus different from “go” stimulus in color; Formdiff go = “go” response to ambiguous stimulus different from “go” stimulus in form.

**Table 4:** Descriptive Statistics on Study Variables Across Four MD GNG Sessions Combined.

	range	min	max	*M* (95%CI)
Ambiguous “go” %	83.34	8.33	91.67	54.31 (47.13; 61.62)
Ambiguous “go” latency	1035.76	791.78	1827.54	1261.70 (1157.27; 1373.85)
Omission error %	50.00	0.00	50.00	15.51 (10.63; 21.55)
Commission error %	33.33	0.00	33.33	11.49 (8.04; 14.94)
Correct “go” latency (ms)	926.00	709.88	1635.88	1116.84 (1025.84; 1211.33)
Commission error latency (ms)	2734.00	266.00	3000.00	1826.87 (1443.50; 2214.62)

*Note*. A one-way ANOVA indicated that mean latencies to correct “go”, commission error, and ambiguous “go” responses differed, *F*(2,84) = 10.122, *p* < 0.001, with LSD follow-up tests indicating a difference between latencies of correct “go” responses and commission errors (*p* < 0.001) and between commission errors and ambiguous “go” responses (*p* = 0.001) but no difference between correct “go” and ambiguous “go” responses (*p* = 0.388).

### Aim 1: Examine whether differences in canine personality is related to *active choice* behaviors reflecting positive/negative expectations about ambiguous stimuli

#### “Go” Responses to Ambiguous Stimuli

Go response % to ambiguous stimuli was predicted by conscientiousness, *F*(1,29) = 5.98, *p* = 0.022 and extraversion, *F*(1,29) = 4.06, *p* = 0.054. Greater go response % to ambiguous stimuli was associated with greater conscientiousness, *β* = 2.45, *p* = 0.022 (SE = 1.00; 95% CIs [0.39; 4.50]) and with greater extraversion, *β* = 1.51, *p* = 0.054 (SE = 0.75; 95% CIs [−0.31; 3.05]). Conscientiousness accounted for 19% and extraversion for 14% in the outcome (*η*_p_^2^ = 0.19 and 0.14, a large and a medium-large effect, respectively^51^). Go response latency was not predicted by any independent or covariate variables (*p*s > 0.305).

### Aim 2: Determine whether results on the association between task performance and ADHD-B/S can be replicated in a modified design

When considering which independent variables were associated with which dependent variables, the results obtained in^32^ are largely consistent with the findings obtained in the current study.

#### Errors

In this study, H/I predicted omission error percent, *F*(1,29) = 9.66, *p* = 0.004, in that greater H/I scores were associated with greater omission error percent, *β* = 1.91, *p* = 0.004 (SE = 0.62; 95% CIs [0.65; 3.18]) and accounted for 26% of the variance in the outcome (*η*_p_^2^ = 0.26; a large effect).

#### Response times

Training status and confidence jointly predicted average latency to correct “go” responses (*χ*^2^(4) = 7.88, *p* = 0.096). Dogs were *less likely* to have an earlier correct “go” response if they had none compared to advanced training (exp(*β*) = 0.18, *p* = 0.037 [0.04; 0.90]) (but the intermediate to none or the basic compared to none difference was not significant). Dogs were *more likely* to have an earlier correct “go” response if they were higher on confidence (exp(*β*) = 1.11, *p* = 0.029 [1.01; 1.22]).

Extraversion predicted average latency to commission errors (*χ*^2^(1) = 5.16, *p* = 0.023). Dogs were *more likely* to have an earlier commission error if they had higher owner-rated extraversion scores (exp(*β*) = 1.17, *p* = 0.020 [1.03; 1.33]).

### Exploratory Aim 1: Determine effect of expanded time window on range of errors

**Figure 3:**
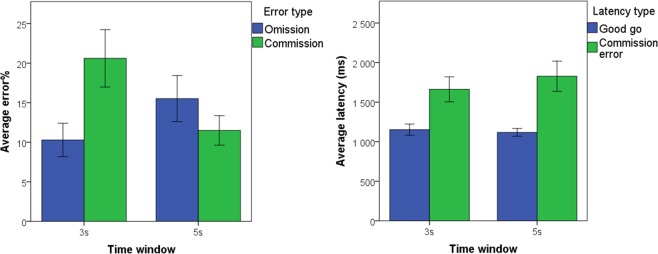
Dogs’ average proportion of errors and response latencies, given error and latency type, across the 3- and 5-s tests. *Note*. Error bars represent 1 SE of the mean. The data reported here for the 3 s window was also reported in (Bunford *et al*., 2018).

For data obtained in^32^ and corresponding descriptive statistics obtained in the current sample, see Fig. 3. There was no difference between the 3 s and the 5 s test in terms of omission error %, average latency of correct “go” responses, or, average latency of commission errors (all *p*s > 0.096) but there was a difference across tests in commission error %, in that dogs exhibited more errors during the 3 s (*M* = 20.60, 95% CIs [13.79; 27.93]) than during the 5 s (*M* = 11.49, 95% CIs [8.04; 14.49]) test, *t*(28) = −2.246, *p* = 0.033.

### Exploratory Aim 2: Evaluate differences in dogs’ behavioral response during uncertainty

**Figure 4:**
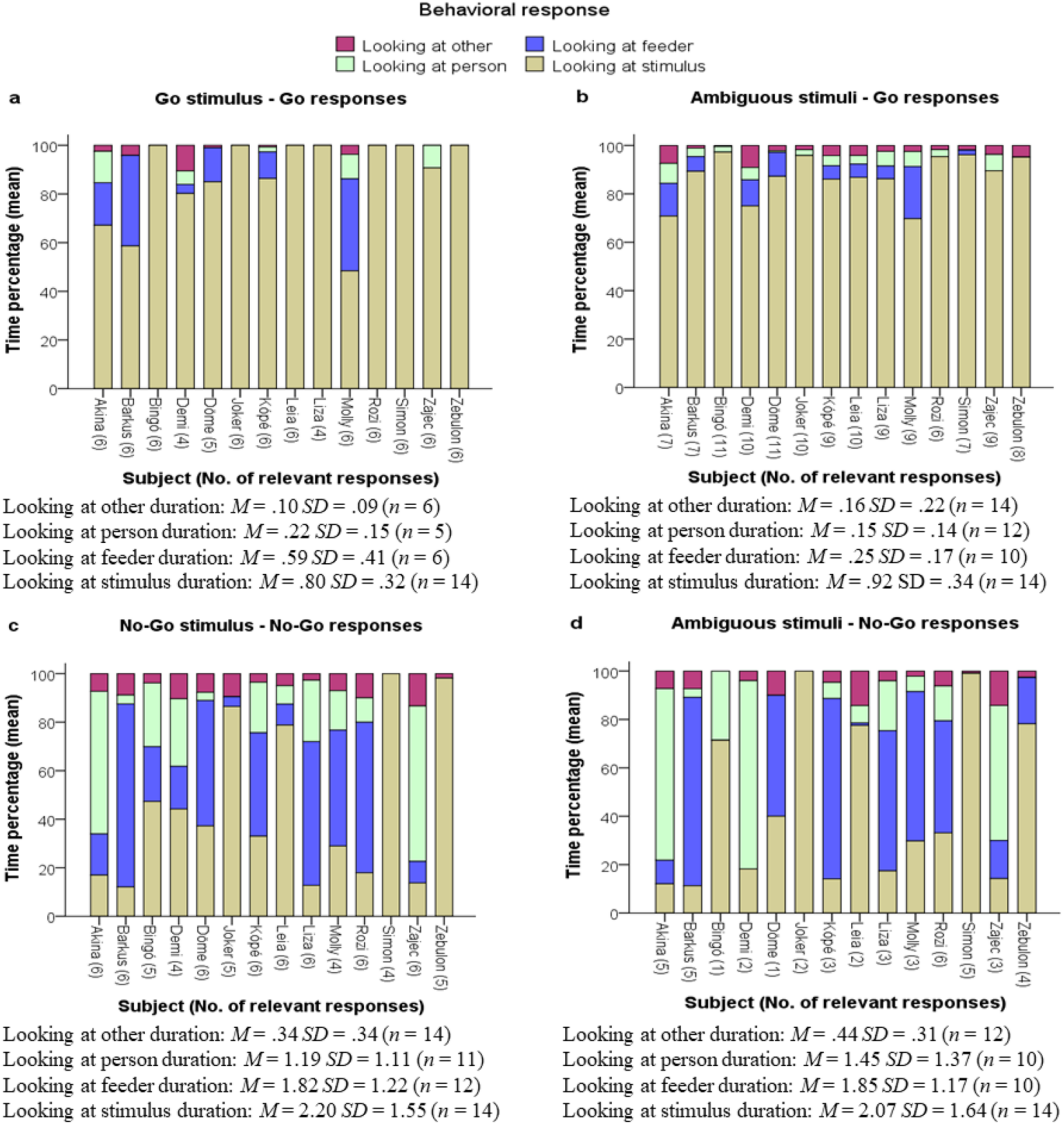
Percentage of time spent using each behavioral approach of interest preceding responses to experimental stimuli. *Note*. *In* the Expanded Time-Window Modified Go/No-Go Test, dogs were presented with 2 sets of 12 stimuli (25% “go”, 25% “no-go”, and 25% ambiguous (in color) and 25% ambiguous (in shape) stimulus). The figures depict, for each dog, the proportion of time during which it executed each of four behavioral approaches of interest in each of four stimulus-response scenarios, i.e., (**a**) a correct “go” response to “go” stimuli, (**b**) a “go” response to ambiguous stimuli, (**c**) a correct “no-go” response to “no-go” stimuli, and (**d**) a “no-go” response to ambiguous stimuli. Behavioral approaches of interest are looking at person (green), the feeder (blue), at the stimulus (light brown) or something other than these (purple). Dogs had 5 s to respond after stimulus onset, and behavioral approaches were calculated for this 5 s time-window, as time-percentage. The numbers in brackets following dogs’ names represent the number of responses executed by the dog to the given stimuli. The descriptive statistics accompanying each of four figure elements represent the average duration (in ss) of each behavioral approach of interest, across dogs, in case of each of four scenarios.

For time-percentages of time spent using each behavioral response preceding “go” and “no-go” responses to ambiguous stimuli, and preceding “no-go” responses to no-go stimuli, see Fig. 4. Upon visual inspection of the data, during the 5 s following ambiguous stimulus onset and before executing a “go” response to such stimuli, all dogs spent most of their time looking at the stimulus on the touchscreen and spent considerably less time looking at the feeder and, on average, even less time looking at their owner or the experimenter, or at something else. Regarding the main pertinent question, that is, whether there are differences between “no-go” responses to ambiguous stimuli and “no-go” responses to no-go stimuli, it appears that dogs did not respond differently to ambiguous stimuli than to the familiar no-go stimuli. (Of note, dogs exhibited a slight bias towards executing a “go” response to ambiguous stimuli over “no-go” responses in the subsample/Expanded time-window GNG test, *t*(13) = −6.607, *p* < 0.001, average difference between observed sample mean and the test value = 23.214, 95%CIs [16.666; 29.166]; but this bias was not present in case of the larger sample/MD GNG Test, *p* = 0.259).

## Discussion

Our primary aims in this study were to examine, in a sample of adult family dogs, whether differences in behaviors consistent with more negative/positive expectations about ambiguous stimuli in a modified canine Go/No-Go paradigm are associated with personality and to determine the extent to which we can replicate, in the MD paradigm but within the same subjects, our earlier results on associations between task performance, personality, and ADHD-B/S. We were also interested in whether expanding the time window during which dogs could execute/withhold a response would increase the range of their errors and wanted to characterize the different types of behaviors dogs engaged in when facing uncertainty about whether to execute or withhold a Go/No-Go response.

Prior to discussion of our findings, a few considerations are prudent. First, earlier animal cognitive bias findings could be conceptualized as pertaining to differences in pessimism/optimism, insofar as there is an inherently emotional and/or hedonistic component to the employed experimental paradigms. In case of our findings, because dogs were rewarded both for correct “go” and for correct “no-go” responses, our cognitive bias results are most accurately interpreted as reflecting differences in *active choice* given positive or negative expectations (as opposed to emotionally or hedonistically-driven pessimism or optimism) about ambiguous stimuli.

It should also be noted that – also applicable to the broader literature, the extensive training required for successful GNG and now MD GNG testing, may confound the results. In addition, our sample was contraselected for less cooperative, skilled, or trained dogs and, as such, is less representative than is the case of most other canine ethological studies. Others have noted that ambiguous stimuli may lose their ambiguity with repeated training and testing, and this issue also requires additional attention^52^.

In the current sample, the conscientiousness subscale of the Canine Big Five Personality Inventory had low internal consistency. We chose to retain the subscale in our analyses. First, conscientiousness is a well-established personality trait in humans, though less is known about it in animals and what is known is based on mixed results. In factor analytic studies of dogs and 11 other animal species, conscientiousness appeared as an independent dimension in humans and chimpanzees but not in dogs^53^. This has contributed to some^53^ arguing that instead of measures adapted for dogs, measured developed specifically for assessing individual differences in personality in dogs should be used when assessing such differences in this species^54–59^. Of note, some traits represented in these latter types of measures are highly relevant to GNG task performance (e.g., responsiveness to training)^56^. Yet others, however, have found acceptable to good internal consistency (α = 0.70) and inter-rater (*r* = 0.81) and test-retest (*r* = 0.47) reliability for the conscientiousness subscale of the Canine Big Five Personality in a large, international sample (*n* = 518)^60^. These across-study differences may be due to differences in sample sizes affecting reliability estimates, though this is difficult to judge as sample size recommendations for reliability analyses vary widely, ranging from a minimum of 15^61^ to 300^62^. Thus, prior to adoption of a four-dimensional model of dog personality excluding conscientiousness as some others have done^30^, additional research on the psychometric properties of the various conscientiousness scales and subscales are necessary. As such, as the current study was exploratory in several aspects, we retained the conscientiousness subscale, but recommend caution in interpreting results involving this subscale.

More generally, although other measures are certainly available, the Big Five questionnaire for dogs has been feasibly used in studies focused on similarities in personality traits between owner-dog dyads^31,63,64^ and also as a tool to assess individual differences in personality and the associations thereof to performance in behavioral paradigms^65^. Further, the Big Five questionnaire was an ideal tool to address our research questions given that our specific aim was to examine the association between dogs’ personality and cognitive bias, indexed via active choice in a GNG paradigm reflecting expectations about ambiguous stimuli. Our general aim was to do so in a manner that our tools and findings can be placed in a proper comparative context and that appropriate comparisons with human findings can be made. With regard to the latter, Big Five personality traits have been shown to be related to differences in behavioral (response) inhibition – ideally probed in GNG tasks – and to ADHD. For example, conscientiousness is negatively linked to behavioral inhibition^33^ and to ADHD^66–68^.

Certainly, as any measurement modality, other-report on rating scales has both advantages and limitations^69^. In case of other measures of personality such as behavioral observation, while those have the advantage of greater experimental control, they have the limitation of being specific to a discrete laboratory challenge or paradigm^70,71^ and are limited to a given period or point in time (and are also less ecologically valid). Conversely, owner-report or rating scale measurement in general may be more subjective, it assesses behavior and thus allows for inferences about personality over time (and are also more ecologically valid, less contrived). Of note, others have established convergent validity of owner-report (of dogs’ personality) using a behavioral test battery^72^. Nevertheless, owner-report will be important to complement in future studies via use of other measurement modalities of personality, such as behavioral observation.

As a general note, we observed no differences in dogs’ tendency to interpret ambiguous stimuli as if those were a “go” or a “no-go” stimulus, as indicated by no trend-level differences in dogs’ tendency to respond to such stimuli by executing or withholding the trained response. Of note, as cognitive biases are often used to indirectly index affective and emotional states, animal research on these phenomena has implications for animal welfare^1^ and – at least insofar as we observed no group-level differences in bias – these findings indicate that the type of active choice task employed in the current study may not be as ideal for probing these types of states as passive discrimination paradigms.

### Aim 1 - Differences in canine personality are related to behaviors consistent with positive/negative expectations about ambiguous stimuli

#### Responses

Findings indicated that greater tendency to execute a “go” response to ambiguous stimuli (i.e., a tendency to respond as if the stimulus was a “go” stimulus as opposed to a “no-go” stimulus) was related to higher conscientiousness and extraversion. Generally, these results are consistent with prior human^2,27–29^ and animal (e.g., captive psittacines [*Amazona amazonica*]^73^; and the domestic pig [*Sus scrofa domesticus*]^74^) findings indicating that judgement is influenced by personality. More specifically, these results are in line with human data that suggest an association between optimism and extraversion^2,27–29^.

We did not observe a relationship between optimism and confidence, unlike in prior animal studies, where cognitive bias (measured as attention bias for environmental stimuli, indexed by the proportion of balks and errors when performing a spatial foraging task) has been shown to relate to neuroticism in captive psittacines (*Amazona amazonica*)^73^. This lack of an effect for confidence is also unlike in prior human studies, where an association between optimism and low neuroticism is consistently observed^2,27–29^. This inconsistency across past and current results may reflect differences across experimental design. Specifically, the referenced past studies involved simpler tests that cognitive bias as in “is hopeful/is not hopeful”. The current study involved an active-choice judgment bias test wherein correctly executed (correct “go”) and correctly withheld (correct “no-go”) responses were rewarded. This difference may account for differences in the degree to which confidence plays a role in task approach, with it playing a larger role in the former type of experimental setup but reinforcement-learning playing a larger role in the latter case.

### Aim 2 – Replicability of earlier results

*Indicating replicability or consistency*, IA did not predict omission or commission errors neither previously nor currently. Earlier, dogs with no training had longer response latencies than dogs with basic training. Here, too, dogs with no training had longer correct “go” response latencies than dogs with advanced training. In our prior *and* current research, dogs were more likely to have an earlier commission error if they had higher extraversion scores.

Conversely, earlier, H/I predicted commission errors and training status predicted commission error latency (none compared to basic training predicted earlier commission errors) but here, these associations were not observed. Indeed, while training status generally played a relatively large role in predicting several of the outcomes in^32^, it was involved in predicting less here, potentially because the additional research-related training and testing “equalized” dogs in this regard.

A few general considerations regarding replicability are worthy of note. First, across-studies comparisons must consider that in our earlier study, 60% of the test stimuli were “go” and 40% were “no-go” stimuli whereas in the current study, these proportions were 50-50. While the former is consistent with the underlying assumption of go/no-go tests (i.e., that the “go” response is “automatic” or “prepotent” by virtue of it being presented *more* than the “no-go” stimulus), the latter is not. As such, the 60-40 design is more ideal for probing behavioral inhibition than the 50-50 design. Indeed, as the proportion of “no-go” stimuli increased from the first study to the current one, the relative proportion of commission errors decreased, indicating that because dogs were presented with the “no-go” stimuli more and the corresponding behavior was practiced more, it required less inhibitory control.

Second, it would make sense for outcomes that are malleable with training and learning to change from our earlier study to the current study and this change would not be incongruent with replicability or consistency. However, the same is not the case for the *relationship* between the variables of interest (i.e., behavioral performance, IA, personality), which should be largely unchanged across studies, if there is replicability and consistency. Taken together, our results and these considerations indicate that the degree to which across-study results converge depend on the outcome considered.

### Exploratory Aim 1

In our prior research, dogs exhibited a relatively narrow range of errors, which may have contributed to us not having observed certain expected associations. Essentially, here, we had one practical question, and that was to determine whether (and the degree to which) allowing for more time to pass between stimulus presentation and feedback would result in greater differences in behavioral performance. As such, this was both a pilot to inform future iterations of the canine GNG paradigm and an attempt to test whether our earlier results were less valid because of a potentially poorly chosen time-window.

Because dogs had more time for a “go” response (more time had passed without them receiving the food reward and/or the wider time window may have resulted in increased difficulty in inhibiting a “go” response), an increase in correct “go” responses and commission errors would have indicated that the 5 s window is more ideal for purposes of examining a range in GNG behavioral performance in dogs than the 3 s window. We did not observe an increase in errors. Rather, we observed a decrease in commission errors, which may be a result of the training dogs received prior to and during modified GNG tests, which may have improved performance more than the expanded time window would have impaired it. Alternatively, it is certainly possible that trait optimism/pessimism affects the likelihood of commission errors as the time window approaches closure regardless of the length of that window, e.g., greater optimism may correspond to greater likelihood of risky strategies (more commission errors) whereas greater pessimism may correspond to greater likelihood of risk averse strategies (more omission errors).

As noted, others have used even longer time windows when testing individual differences in cognitive bias (e.g., 10 s in Starling *et al*.^21^ and 30 s in^19^). Nevertheless, we argued that extending the time-window to such lengths in the current study would have been impractical, suggesting that doing so would have resulted in there being only or almost only commission errors and no or almost no omission errors. Our current results seem to suggest the opposite.

### Exploratory Aim 2

Regarding behavioral response to uncertainty about how to respond, before executing a “go” response to ambiguous stimuli, all dogs spent most of their time looking at the stimulus. Dogs’ behavioral response patterns to “go” stimuli were different than to ambiguous and “no-go” stimuli, which did not differ from each other. We expected that dogs, when facing increased uncertainty in case of the ambiguous stimuli, would rely more on social reference (i.e., to look more at their owner or the experimenter) than when presented with a familiar “no-go” stimulus. This was not the case. Rather, it appears that dogs were tuned into a “to inhibit or not inhibit” cognitive set, where once their reaction was to inhibit, they withheld their behavioral response both to ambiguous and to trained “no-go” stimuli. It may be that the proportion of time dogs spent looking at their owners in response to trained “no-go” stimuli is comparable to the proportion of time they spent looking at their owners in response to ambiguous stimuli because both inner states of inhibition and uncertainty elicit this behavior. However, these differences in inner states may not be separable based on observable behavior. Dogs may look at their owners, in case of inhibition, for confirmation or reinforcement and in case of uncertainty, for information^75^. In addition, as marked and relatively consistent within-dog variability in case of “no-go” responses was also observable, it appears that dogs followed different strategies during inhibition. As such, dogs are heterogeneous in this regard: When facing uncertainty, those who tend to look at the screen/stimuli appear to behave independently, similarly to wolves^39^, whereas those who tend to look at the feeder seem to exhibit simple reinforcement-seeking behavior, and those who tend to look at their owners are relying more on social reference. These within-species differences may be related to individual differences in attachment or personality, with these associations potentially emerging in larger samples in future studies.

Further, during Pavlovian conditioning, if a cue predicts reward in a different location, some human and nonhuman animals will approach the site of reward, i.e., engage in goal tracking whereas others will approach and interact with the cue, i.e., engage in sign tracking^76^. Individual differences in these regards may have had an effect on dogs’ behavioral responses while they withheld the prepotent action in response to “no-go” stimuli and when they appeared uncertain about how to respond to such stimuli. Although our experimental design was not intended to assess the effect of such nuances, examination of their effects is a potential avenue for future research.

### Limitations and future directions

Although us having obtained data generally confirming our hypotheses speak to the robustness of the observed effects, arguably, even greater confidence could be placed in these findings and their generalizability if replicated with a larger sample. As such, the authors of future studies are encouraged to aim to assess our current research questions with a larger sample of animals, perhaps in multi-site studies so as to also attend to competing needs (e.g., feasibility) of these types of studies.

Despite their noted advantages, there are also difficulties with and limitations of the type of GNG tasks employed in this study. Specifically, considerable pre-training is necessary and the paradigm may not be useable with dogs at extreme ends of certain continua, such as the ADHD-like behaviors/symptoms continuum. Conversely, despite their noted limitations, the more traditional (simple discrimination) cognitive bias tests are easier to use and are useable with a wider range of animals. Furthermore, as in humans, the type of GNG tasks employed in this study are certainly probing differences in characteristics beyond cognitive biases, such as in individual differences in attention and behavioral disinhibition.

Examining more complex relationships among variables than the ones tested here, such as interactions, will be an important next step to better understand these results. For example, earlier and replicated in the current study, greater extraversion was associated with more commission errors and in the current study, greater extraversion was associated with greater tendency to execute a “go” response to ambiguous stimuli (i.e., greater optimism). It may be a reasonable next step in this line of research to experimentally disentangle the effect of extroversion and optimism on behavioral performance, as experimental manipulation of optimism may result in different behavioral responses in individuals high than in individuals low on extroversion.

## Conclusion

Summarizing key aims and results, the current study is the first to establish a relationship between a behavioral index of more positive/negative judgments about ambiguous stimuli in a modified Go/No-Go paradigm and individual differences in personality in domestic dogs. Our findings add to a growing body of research by extending the dog as an animal model of human behavior and cognition to the association between an optimistic cognitive bias and personality.
